# Veterinary Medical Association of New Jersey

**Published:** 1900-02

**Authors:** S. Lockwood

**Affiliations:** Secretary


### VETERINARY MEDICAL ASSOCIATION OF NEW JERSEY.

THE sixteenth annual meeting was held at 495 Broad Street, Newark, January 11, 1900. The meeting was called to order by the President, Dr. L. P. Hurley, at 11 o'clock. Ten members were present at roll-call, and about twenty applicants for membership, also two delegates from the Keystone Association—Drs. W. Horace Hoskins and W. L. Rhoads, and Dr. L. Pearson, President of the American Veterinary Medical Association.

Minutes of previous meeting read, and, after correction, approved. The President then made his annual address, and the Secretary's report was read and accepted.

Dr. Wm. Herbert Lowe then read the following resolutions: *Whereas*, the Veterinary Medical Association of New Jersey has learned with profound sorrow of the death of Dr. Wm. H. Arrowsmith, of Jersey City, former President of this Association; *whereas*, Dr. Arrowsmith was held in high esteem, not only by the members of this Association, but by the profession throughout the State: *and whereas*, Dr. Arrowsmith during his entire professional career maintained the dignity of the profession, always doing his share to promote the elevation of veterinary science and art; therefore, be it *resolved*, that we record these facts, and that the profession has sustained a great loss in his death, and that we, the members of the Veterinary Medical Association of New Jersey, a personal friend and associate, whose memory shall long remain fresh in our recollections; and be it further *resolved*, that these resolutions be spread in full upon the minutes and a copy be sent to the family of the deceased. Wm. Herbert Lowe, J. W. Hawk, and James D. Hopkins, Committee. Adopted.

Dr. Lowe then read the agreements of the New Jersey State Veterinary Society and Veterinary Society of the State of New Jersey to merge into and join the Veterinary Medical Association of New Jersey.

The President then ordered a recess for half an hour for the board to make their report, and at 12 o'clock the Board of Censors reported favorably on the following applicants for membership: Drs. Edwin Ancker, 167 West Thirty-first Street, New York; A. W. Axford, Naughwright; F. E. Brooks, 237 Seventeenth Avenue, Paterson; A. Brown, Windsor; E. Buckley, East Orange; T. Earle Budd, 90 Part Street, Orange; J. J. Cattanach, Morristown; J. C. Corlies, 29 East Park Street, Newark; T. J. Cooper, 5 Bank Street, Paterson; Wm. Dimond, 95 Summit Street, Newark; W. H. Dustan, 29 De Hart Street, Morristown; Van. C. Dull, 104 Cross Street, Paterson; A. D. Edwards, Atlantic Highlands; G. P. Ellice, Morristown; R. W. A. English, Boulevard and Communipaw Avenues, Jersey City; J. M. Everitt, Hackettstown; Hugh Exton, Washington; W. C. Ferguson, 589 Broadway, Paterson; J. B. Finch, Ramsey; W. J. Fredericks, Delawanna; Wm. Gall, Matawan; J. O. George, Camden; Julius Gerth, Newark; James T. Glennon, 146 Summer Avenue, Newark; G. F. Harker, 147 Perry Street, Trenton; W. F. Harrison, Bloomfield; R. O. Hasbrouck, 160 Jefferson Street, Passaic; J. W. Hawk, 29 Halsey Street, Newark; Howard S. Holdenby, Ridgewood; Edwin A. Hogan, 24 Plane Street, Newark; Jas. D. Hopkins, 39 Orchard Street, Newark; J. B. Hopper, Ridgewood; L. P. Hurley, Hopewell; Nicholas J. Kaiser, 52 Niagara Street, Newark; John Kehoe, Lyndhurst; B. F. King, Little Silver; M. O. M. Knott, 159 Crescent Avenue, Plainfield; Henry J. Lehrmann, Montclair; Otto R. Leis, 24 Frederick Street, Newark; E. L. Loblein, New Brunswick; S. Lockwood, Woodbridge; Wm. Herbert Lowe, 188 Ellison Street, Paterson; J. Payne Lowe, 185 Jefferson Street, Passaic; Anthony P. Lubach, Pompton Lakes; A. Machan, 58 River Street, Paterson; Chas. T. Magill; Haddonfield; E. Mathews, 307 Second Street, Jersey City; James T. McDonough, Montclair; P. F. McGuiness, 77 Park Street, Newark; A. H. McIntosh, Summit; W. B. E. Miller, Camden; John M. Mitchell, Madison; J. Mosedale, Morristown; E. R. Ogden, Orange; George W. Pope, U. S. Quarantine Station, Garfield; H. W. Reed, Freehold; W. J. Reagan, 606 River Street, Paterson; J. G. Richardson, Morristown; W. Runge, 130 Union Street, Newark; L. R. Sattler, 22 Seventeenth Avenue, Newark; J. Serling, 310 West 125th Street, New York; Arthur W. Smith, Bloomfield; Raymond B. Smith, Montclair; T. E. Smith, 309 Barrow Street, Jersey City; S. S. Treadwell, Englewood; H. Van de Roest, 158 Orchard Street, Newark; A. G. Vogt, 121 Plane Street, Newark; E. R. Voorhees, Somerville;-F. A. Zucker, 20 West Jersey Street, Elizabeth. All were unanimously elected; the board also reported favorably on accepting the offer of the other societies.

Dr. W. H. Lowe then offered the following resolution: *Whereas*, the New Jersey State Veterinary Society at a special meeting held at the residence of Dr. James D. Hopkins, President, 39 Orchard Street, Newark, N. J., January 5, 1900, for the purpose of merging that society with this association, did unanimously pass a resolution agreeing to cease to exist as an independent organization, and did further agree to merge and join with this association; therefore, be it *resolved*, that the Veterinary Medical Association of New Jersey agree to and do hereby accept the proposition of the New Jersey State Veterinary Society, merging the said society with this association, admitting such members as are in good standing, whose dues are paid in full to date, without initiation fees, with full rights and privileges as are now enjoyed by members of this Association, upon the said society passing over all books, papers, and other property to the Veterinary Medical Association of New Jersey.

Dr. James D. Hopkins, last President of the New Jersey State Veterinary Society, then turned over the charter and books of their Society to Dr. L. P. Hurley, President of this Association, who accepted them and made a short address of welcome, and the merging of the New Jersey State Veterinary Society into the Veterinary Medical Association of New Jersey was consummated. Adjourned at 1.15 for dinner; reconvened at 2.30.

*Whereas*, the Veterinary Society of the State of New Jersey and Vicinity, at a special meeting held at the residence of President Dr. L. R. Sattler, No. 22 Seventeenth Avenue, Newark, N. J., on Thursday, December 28, 1899, for the purpose of merging that Society with this Association, did unanimously pass a resolution, agreeing to cease to exist as an independent organization, and did further agree to merge and join with this Association; therefore, be it *resolved*, that the Veterinary Medical Association of New Jersey agree to and do hereby accept the proposition of the Veterinary Society of the State of New Jersey and Vicinity, merging the said Society with this Association, admitting such members as are in good standing, whose dues are paid in full to date, without initiation fees, with full rights and privileges as are now enjoyed by members of this Association, upon the said Society passing over all books, papers, and all other property to the Veterinary Medical Association of New Jersey.

Dr. L. R. Sattler, President of the Veterinary Society of the State of New Jersey and Vicinity, and Dr. E. Buckley, the Secretary, were present and surrendered the books and charters and papers to the President, who welcomed them into the fellowship of the only Veterinary Medical Association of New Jersey.

Dr. William Herbert Lowe then made an address, followed by Dr. Leonard Pearson, Dr. W. Horace Hoskins, and Dr. W. L. Rhoads, of Pennsylvania, and by several members of the Association; Dr. T. Earle Budd read a paper on "Maternal Dystocia," but being late there was no discussion.

Dr. Lowe offered the following resolution: *Resolved*, that the Veterinary Medical Association in annual convention assembled hereby approves and indorses the plan to organize an Army Veterinary Contingent of Commissioned Veterinarians under a chief veterinarian having adequate rank; and be it further resolved, that the members of this Association earnestly request the members of Congress from New Jersey, and especially the Senators—the Hon. W. J. Sewell and the Hon. John Kean—to use their influence and all honorable methods to advance the above plan.

The Secretary was instructed to have typewritten copies sent to each Senator and Congressman in the State, with the names of all members attached.

Moved and carried to meet in Newark in May, the place to be selected by the President and Secretary. Adjourned at 4.15.

S. Lockwood,
Secretary.

